# Forced displacement and gendered violence in conflict zones in the southern Philippines: the experiences of internally displaced people from Zamboanga and Marawi

**DOI:** 10.3389/fsoc.2026.1655806

**Published:** 2026-09-03

**Authors:** Diana Therese Montejo Veloso

**Affiliations:** Department of Sociology and Behavioral Sciences, De La Salle University, Manila, Philippines

**Keywords:** forced displacement, involuntary migration, gender-based violence, internally displaced people, armed conflict, ethnoreligious conflict, Zamboanga Siege, Marawi Siege

## Abstract

Migration is not always by choice, but rather, influenced by circumstance. Displacement due to human-made disasters, such as armed conflict, constitutes forced migration, also known as involuntary migration. This paper examines the experience of forced displacement due to armed conflict and the risk of gender-based violence (GBV) in conflict zones in the southern Philippines, focusing on the survivors of the 2013 Zamboanga Siege and the 2017 Marawi Siege. The researcher examines the dynamics of violence during armed conflict from a gendered perspective and illuminates the extent to which gender-based violence exists on a continuum from personal, to community based and/or state-sponsored violence. Drawing upon interviews and focus group discussions with internally displaced people (IDPs) in traditional evacuation centers, home-based evacuation arrangements, and/or transitory sites, and with duty-bearers from Zamboanga City and the Islamic City of Marawi, this paper discusses the pathways to evacuation of IDPs from both locations and how these shaped the experiences of GBV among survivors. The researcher identifies common themes and nuances in experiences of personal, community, and state-sponsored gendered violence among women and girls and men and boys, who experienced involuntary migration through forced displacement. Migration is not always by choice, but rather, influenced by circumstance. Displacement due to human-made disasters, such as armed conflict, constitutes forced migration, also known as involuntary migration. This paper examines the experience of forced displacement due to armed conflict and the risk of gender-based violence (GBV) in conflict zones in the southern Philippines, focusing on the survivors of the 2013 Zamboanga Siege and the 2017 Marawi Siege. The researcher examines the dynamics of violence during armed conflict from a gendered perspective and illuminates the extent to which gender-based violence exists on a continuum from personal, to community based and/or state-sponsored violence. Drawing upon interviews and focus group discussions with internally displaced people (IDPs) in traditional evacuation centers, home-based evacuation arrangements, and/or transitory sites, and with duty-bearers from Zamboanga City and the Islamic City of Marawi, this paper discusses the pathways to evacuation of IDPs from both locations and how these shaped the experiences of GBV among survivors. The researcher identifies common themes and nuances in experiences of personal, community, and state-sponsored gendered violence among women and girls and men and boys, who experienced involuntary migration through forced displacement. This paper compares and contrasts the extent of armed conflict and the trends in GBV among forced migrants in both cities. The researcher exposes the incidences of gendered violence experienced by IDPs in Zamboanga, the vulnerability of women and children to domestic violence and trafficking, and the covert attempts to recruit men into extremist groups. Moreover, this paper looks into trends in extremist violence, militarism, and other forms of gendered violence among IDPs from Marawi and the risk of clan feud upon returning to their communities. The researcher highlights the links between gender, racial, ethnic, religious, and social class inequality in the Philippines and the vulnerability of people displaced by armed conflict. This paper delves into the continuity of violence at various levels and assesses the intersections between private and public violence confronting IDPs from Zamboanga and Marawi.

## Introduction

People relocate within or across national borders through diverse pathways. Yet migration is not always by choice, but rather, influenced by circumstance. Forced migration, also known as involuntary migration, has various driving forces, but typically involves “force, compulsion, or coercion” (International Organization for Migration [IOM] 2019, cited in Migration Data Portal, 2024, para. 2). This manifests during displacement, defined as the relocation of people due to armed conflict, violence, human rights violations, and natural or human-made disasters (IOM 2019, cited in Migration Data Portal; International Organization for Migration, 2024; United Nations, 2025a).

According to the United Nations (2025a), forced displacement constitutes involuntary migration: “Others move to escape conflict, persecution, terrorism, or human rights violations” (para. 1). Internally displaced persons (IDPs), also called “internal migrants” (International Organization for Migration, 2025, para. 1), comprise smaller proportions of migrants, who, however, frequently need assistance. This characterizes the situation of people displaced by armed conflict in the southern Philippines, particularly the 2013 Zamboanga Siege and the 2017 Marawi Siege. These insurgencies in the western and northern regions, respectively, of Mindanao, were perpetuated by militant separatist, extremist, and/or Islamist groups, leading to the displacement of numerous civilians, who are often racial, ethnic, and religious minorities. Gender-based violence has become prevalent in conflict and post-conflict zones in Mindanao (Veloso, 2022a; Veloso, 2022b). Security remains a paramount concern, as is the lack of normalization in IDPs’ lives after armed conflict.

This paper highlights the lived experience of forced displacement and examines the risk of gendered violence and other security concerns affecting IDPs from the cities of Zamboanga and Marawi. This research examines the trends in their experiences of victimization in conflict and post-conflict situations and issues affecting their well-being.

### Armed conflict and gendered violence

Survivors of forced displacement are vulnerable to gendered victimization during and after armed conflict (Migration Data Portal, 2024; Simeon, 2023). Research underscores the heightened risk of gender-based violence in conflict and post-conflict situations across different countries (Giles and Hyndman, 2004; Human Rights Watch, 2006a; Human Rights Watch, 2006b; Lee-Koo, 2011; Rawi, 2004; United Nations Development Fund for Women, 2003; World Health Organization, 2010). Gender-based violence (GBV) refers to harmful acts directed against individuals or groups based on their gender (United Nations Office of the High Commissioner for Human Rights, 2014). Gender pertains to the social and cultural construction of behaviors, roles, identities, and statuses associated with masculinity and femininity (Lorber, 2005; West and Zimmerman, 1987). Violence includes physical, written, or verbal actions that inflict, threaten, or cause injuries that are physical, psychological, material, or social in nature (Jackman, 2002; World Health Organization, 2006; World Health Organization, 2010).

Gendered violence encompasses, but is not limited to: (1) physical, sexual, and psychological violence within the family, including battering, marital rape, sexual abuse of children in the household, harmful traditional practices, and violence related to exploitation; (2) physical, sexual, and psychological violence in the community, including rape, sexual abuse, intimidation at work, in educational institutions and the like, trafficking in women, and prostitution; and (3) physical, sexual, and psychological violence perpetuated or condoned by the state, wherever it occurs (UN Declaration on the Elimination of Violence Against Women, cited in United Nations Development Fund for Women, 2003). Research reveals that GBV exists on a continuum, which includes private and public violence (Aulette and Wittner, 2011; Lorber, 2005; Radford and Stanko, 1996; United Nations Development Fund for Women, 2003); this continuum also includes interpersonal and structural violence (Hourani et al., 2021; Phillimore et al., 2025; Sisic et al., 2025). Gender, sexuality, and other interconnected social positions, are weaponized in acts of violence to assert power (Human Rights Watch, 2007; Medica Mondiale, 2025; United Nations Office of the High Commissioner for Human Rights, 2025). This applies to survivors of forced displacement.

Violence against women during war and conflict persists in many nations (Amnesty International, 2003; Amnesty International, 2005; Human Rights Watch, 2006a; Human Rights Watch, 2006b; Lee-Koo, 2011; Marshall, 2006). Women bear the brunt of gendered and sexualized violence by enemy and “friendly” forces alike (AFP, 2005; Human Rights Watch, 2000; United Nations Development Fund for Women, 2003; Wali et al., 1999; World Health Organization, 2006). Rape and other forms of sexual assault against women are an integral part of armed conflict, as women are deemed the “spoils of war” (Human Rights Watch, 2000; Medica Mondiale, 2025; United Nations Office of the High Commissioner for Human Rights, 2025; Rawi, 2004). In patriarchal cultures, women are treated as the property of men (Green, 1999; Marshall, 2006). Rape and other forms of sexual victimization against women are weaponized to attack men of particular ethnic groups, religions, or nationalities during wartime (Caiazza, 2004; Goldstein, 2001; Human Rights Watch, 2007; World Health Organization, 2006). Women, as well as girls, have been abducted and made into sex slaves, “comfort women,” or “wives” of the enemy (Caiazza, 2004; Human Rights Watch, 2006b; Human Rights Watch, 2007). They have been sold into prostitution by their families or have engaged in it for survival during and after armed conflict (Marshall, 2006; National Law Enforcement Coordinating Committee Sub-Committee on Organized Crime, 2015; National Law Enforcement Coordinating Committee Sub-Committee on Organized Crime, 2016; World Health Organization, 2006).

Men experience gendered violence in wartime through rape, forced intercourse with and/or witnessing the rape of women family members or relatives, forced intercourse with men prisoners, and torture (Enloe, 2004; Human Rights Watch, 2007; Lee-Koo, 2011). They also face the risk of organ trafficking and forced membership in extremist groups (National Law Enforcement Coordinating Committee Sub-Committee on Organized Crime, 2015; Veloso, 2022a).

Children are vulnerable to gender-based violence during conflict and its aftermath (Human Rights Watch, 2006b; United Nations Office of the High Commissioner for Human Rights, 2025; United Nations Children’s Fund [UNICEF], 2022; United Nations, 2025b; Veloso, 2022a). The United Nations Security Council (cited in United Nations Children’s Fund, 2024) highlighted six serious violations against children during wartime, namely: killing and injuring children, recruitment or deployment by armed forces or armed groups, rape or other serious acts of sexual violence, abduction, attacks on schools and hospitals—whether or not they are directly targeted—that compromise their well-being, and the denial of humanitarian assistance and access. Girls are predominantly victimized by rape and other acts of sexual violence as a wartime strategy (Human Rights Watch, 2006b; United Nations Children’s Fund, 2022); they are also vulnerable to abduction, forced marriage, torture, and arbitrary killings (Human Rights Watch, 2007; United Nations Office of the High Commissioner for Human Rights, 2025). Boys are frequently targeted for abduction, motivated by recruitment and utilization by armed groups, and killing and maiming (United Nations Children’s Fund, 2022); they also endure sexual violence in detention facilities (United Nations Office of the High Commissioner for Human Rights, 2025), and sexual harassment or other forms of nonconsensual sex with men (Goldstein, 2001; Veloso, 2022a) in conflict zones. Children experience trauma and developmental issues after witnessing domestic violence and the attack and/or killing of their parents (Medica Mondiale, 2025; Wali et al., 1999).

## Contextualizing armed conflict in Zamboanga and Marawi

### Zamboanga Siege: an overview

On September 9, 2013, fighting broke out in Zamboanga City and continued until September 28, 2013 (Sinapit, 2013). A faction of the Moro National Liberation Front (MNLF) perpetrated the Zamboanga Siege; the MNLF, formed in 1970, was the first Muslim separatist organization that advocated the formation of Mindanao as a distinct state from the Philippines, but signed a peace agreement with the government in 1996 and settled for the autonomy of selected provinces in Mindanao (Abinales, 2008; Abinales and Amoroso, 2005; McKenna, 1998). Civilians and experts attribute the insurgency to two causes. One factor is the refusal of then-Mayor Maria Isabelle Climaco to allow the MNLF faction to raise its flag on its founding anniversary, citing the unconstitutionality thereof. Another motive is the MNLF response to its exclusion in the peace process between the government and the Moro Islamic Liberation Front (MILF). For context, the MILF broke off from the MNLF in 1978 and became the largest separatist organization, but engaged in peace negotiations with the government before the siege; the peace agreement was finalized in March 2014, in exchange for the expansion of the Muslim-majority autonomous region in Mindanao^1^ (Council on Foreign Relations, n.d; Esguerra and Burgonio, 2014).

The MNLF used 194 civilians as human shields; most hostages escaped or were rescued. Moreover, 32 people—including 9 civilians, 5 police officers, and 18 soldiers—were killed, and 238 people—including 57 civilians, 14 police officers, and 167 soldiers—were injured (Santos Jr, 2024; Sinapit, 2013). Coastal villages were hit hard. The residents, who were mostly low-income, informal settlers and racial, ethnic, and religious minorities, fled to avoid the crossfire. At least 10,000 houses in coastal communities were destroyed or damaged (United Nations Office for the Coordination of Humanitarian Affairs, 2014). Some claimed MNLF insurgents were the culprit, while others maintained that the Philippine military burned homes turned into rebel strongholds (Santos Jr, 2024; Veloso, 2022a).

About 118,000 people were displaced for prolonged periods, residing in tents and bunkhouses at the Joaquin F. Enriquez Sports Complex, also known as the Grandstand, or across the facility, along Cawa-Cawa Boulevard (United Nations Office for the Coordination of Humanitarian Affairs, 2014). These numbers do not include people who opted for home-based evacuation. The IDPs at Cawa-Cawa Boulevard left in November 2014, while those at the Grandstand left in July 2015, relocating to transitory sites and in some cases, permanent housing. Numerous IDPs remained in transitory sites or other temporary housing for many years after the siege (Philippine News Agency, 2016; SunStar Philippines, 2017).

### Marawi Siege: an overview

On May 23, 2017, fighting broke out in the Islamic City of Marawi. The military launched operations against Abu Sayyaf Group (ASG) and Maute Group rebels, as well as Islamic State of Iraq and Syria (ISIS) fighters (Dizon, 2017; Hincks, 2017; Maitem, 2017; Marcelo, 2017). Despite claiming to fight for a sovereign Islamic state, the ASG is notorious for bombings, extortion, and kidnappings that have victimized Filipinos and foreign nationals since the 1990s (Abuza, 2005; Alberto and Guinto, 2008; Associated Press & Philippine Daily Inquirer, 2014; Atkinson, 2012; Banlaoi, 2010; BBC News, 2013; Fonbuena, 2014; The Manila Times, 2009; Zamora, 2001). Meanwhile, the Maute Group has perpetuated kidnappings and bombings in Lanao del Sur from 2015 onward (Terrorism Research and Analysis Consortium, 2017; Unson, 2017). Both groups have pledged allegiance to the Islamic State of Iraq and Syria (ISIS), also called the Islamic State (IS), which allegedly eyed Marawi as a potential *wilaya* (province) in its attempt to form a caliphate, a form of Islamic government (Fonbuena, 2017; Francisco, 2017; Hincks, 2017; International Crisis Group, 2018; The Manila Times, 2017).

ISIS rebels took over Amai Pakpak Medical Center, attempted to control Camp Ranaw military base, and set fire to Marawi City Jail and Dansalan College (CNN Philippines, 2017; Hincks, 2017; Marcelo, 2017). Citing rebellion, then-President Rodrigo Duterte declared Martial Law in Mindanao and extended it until 2019 (Diola, 2017; Fonbuena and Bueza, 2017; PhilStar.com, 2017; Rood, 2017). Ten days after the conflict began, 175 people—including 120 extremists, 36 soldiers, and 19 civilians—were killed in military-initiated airstrikes and a “fog of war” or “friendly fire” incident (Agence-France Presse 2017; Placido, 2017; The Guardian, 2017). The Marawi Siege lasted for five months; a ceasefire was declared on October 23, 2017 (Aben, 2018; Bueza, 2017).

To avoid the crossfire, numerous civilians evacuated to other areas in northern Mindanao, such as: Iligan City and the municipalities of Balo-i and Kauswagan in Lanao del Norte province; the municipalities of Saguiaran, Marantao, and Kapatagan in Lanao del Sur province, and Cagayan de Oro City in Misamis Oriental province. Some families transferred to provinces in Central, South-Central, and Southern Mindanao, such as Bukidnon, Cotabato, and Davao (Arguillas, 2017; Estremera, 2017; Lagsa, 2017). Others relocated to provinces in the Visayas, such as Cebu, Negros Oriental, and Leyte (Featuresdesk, 2017; Padayhag, 2017; SunStar Philippines, 2017). Some moved to Metro Manila and provinces in Luzon (Aben, 2018; Arguillas, 2017). According to government reports, IDPs were scattered across at least 87 evacuation centers in the country, as of July 2017 (Cabato, 2017); this number excludes home-based evacuees. As of August 2017, an estimated 359,680 people had been displaced by the Marawi Siege (United Nations Office for the Coordination of Humanitarian Affairs, 2017). Over 120,000 people remained displaced for years after the siege, staying at transitory sites or other temporary housing; many could not return home due to restrictions (Aben, 2018; United Nations High Commissioner for Refugees, 2020; Westerman, 2020).

### Research questions

This paper identifies the pathways to the evacuation of internally displaced people from Zamboanga and Marawi and how these shaped the experiences of gender-based violence among survivors. It assesses the risk of gendered violence impacting IDPs from both cities during and after armed conflict. In particular, this paper compares and contrasts the sieges and the trends in GBV among forced migrants in both cities. This research examines how IDPs understand or articulate their experiences of gender-based violence, delving into how victims or survivors frame GBV and its risk. This paper also examines how gender interacts with other social positions in shaping IDPs’ experiences of gendered violence.

This study seeks to answer the following questions:

1. What were the pathways to evacuation of internally displaced people (IDPs) from Zamboanga and Marawi and how did they shape the experiences of gender-based violence (GBV) among survivors?
2. How has the experience and risk of gender-based violence affected IDPs from Zamboanga and Marawi? In particular, how does gender interact with other social locations, particularly race, ethnicity, and social class, to influence the experiences of IDPs of gender-based violence?

### Theoretical framework

The researcher utilized Crenshaw, (1991) intersectionality theory, which asserts that gender overlaps with other social locating factors, such as race and ethnicity, nationality, social class, religion, sexuality, age, and dis/ability, in shaping people’s experiences of privilege and oppression. As people may be privileged on the basis of certain social positions yet marginalized on the basis of others, this impacts their experiences of gendered violence, treatment by society, and access to safety nets (Hill Collins, 1990; Davis, 1983; Davis, 1984; Hooks, 2000; Lerner, 1986; Radford and Stanko, 1996; World Health Organization, 2006). Their interconnected identities affect the responses of the police, the state, professionals, and the voluntary sector (Barbee and Little, 1993; Hill Collins, 1990; Disch, 2009; Green, 1999; Hester et al., 1996). Intersectionality informs the patterns and nuances in IDPs’ lived experiences and vulnerabilities in conflict zones. The researcher analyzed how IDPs’ intersecting statuses, such as gender, sexual orientation, ethnicity, religion, social class, age, and nationality, shaped their pathways to evacuation, experiences of gendered violence and other safety risks, and access to assistance and support systems during and after displacement. This study examined how IDPs from Zamboanga and Marawi opted for certain evacuation arrangements, perceived gendered violence and security risks, and mobilized—or were deprived of—resources and safety nets, depending on their positionality.

Feminist theory illustrates that GBV spans a continuum of private and public violence, including: personal violence between intimate partners, acquaintances, and strangers; and community-based and/or state-sponsored violence during war and conflict (Aulette and Wittner, 2011; Lorber, 2005; Radford and Stanko, 1996; United Nations Development Fund for Women, 2003). The continuum of GBV also includes interpersonal and structural violence (Hourani et al., 2021; Phillimore et al., 2025; Sisic et al., 2025). The researcher examined how the continuum of gendered violence manifested in IDPs’ experiences of private and public violence and interpersonal and structural violence, be it chronological acts of violence that preceded armed conflict or ongoing acts of violence.

This study applied intersectional feminist theory to analyze the dynamics of gendered violence in conflict and post-conflict situations in Mindanao and the responses of IDPs. Intersectional feminism asserts that gender-based violence is intertwined with patriarchy and the culture of violence in different societies (Kelly, 1996; Steinem, 2004). While the gender dimensions of violence should be assessed in relation to men’s social and economic dominance over women and traditional views on masculinity and femininity, intersectionality informs people’s experiences of gender-based violence and responses to it (Crenshaw, 1989; Disch, 2009; Hooks, 2000; Sheffield, 2004). The researcher examined how IDPs’ overlapping identities shaped their experiences of gender-based violence during and after displacement, their individual and collective responses, and their (in)ability to seek assistance.

## Materials and methods

The research on IDPs in Zamboanga was conducted in 2014 to 2016 and during a follow-up visit in 2019. The research on IDPs from Marawi was conducted in 2017. The researcher observed ethical standards and protocols while collecting data. After obtaining research ethics clearance, the researcher coordinated with heads of government agencies, such as the Municipal Social Welfare and Development Office (MSWDO) in Zamboanga, traditional evacuation centers in Zamboanga and Iligan, and a non-government organization (NGO) in Iligan, before collecting data. The researcher obtained informed consent from the research participants—and in the case of minors, proxy consent from parents, guardians, or other caregivers. The identities of informants were kept confidential.

The researcher utilized qualitative methodology, particularly key informant interviews (KIIs) and focus group discussions (FGDs), to examine IDPs’ pathways to evacuation and common themes and nuances in their accounts and perspectives on gendered violence in conflict zones (see Table 1). This approach fleshed out their narratives about their lived experiences of forced displacement, the risk of GBV, and security issues in times of conflict and/or post-conflict. To obtain firsthand information, the researcher interviewed IDPs who experienced violence during or after armed conflict, and “duty-bearers,” defined as people who provided services to IDPs, be it as paid staff or volunteers. The informants were identified through convenience sampling and snowball sampling, particularly through referrals from duty-bearers^2^ and/or other interviewees.^3^

**Table 1:** Overview of interviews and focus group discussions conducted with IDPs from the cities of Zamboanga and Marawi, and duty-bearers serving IDPs.

Interviews and FGDs conducted	From Zamboanga City		From Marawi City (but residing in nearby cities and municipalities)	
Number of interviews with survivors of gender-based violence (GBV)	35		25	
Number of interviews with duty-bearers serving IDPs	8		Duty-bearers only	Both survivor of GBV and duty-bearer
			4	13
Number of FGDs with IDPs	FGDs at traditional evacuation center	FGDs at transitory sites	FGDs at traditional evacuation center	FGDs at home-based evacuation communities
FGDs with parents	4	8	2	2
FGDs with youth	4	8	2	2
Number of FGDs per type of evacuation and/or temporary housing arrangement	8	16	4	4
Total number of FGDs conducted with IDPs	24		8	

The informants from Zamboanga included 35 IDPs. The researcher also conducted informal interviews with eight duty-bearers in Zamboanga, particularly the Women-Friendly Space (WFS) Facilitators, Peacekeepers, Job Order Staff, and the Camp Manager. Meanwhile, the informants from Marawi, who resided in nearby cities and municipalities, included 25 IDPs, of whom 13 were both IDPs and duty bearers, and four duty-bearers, who worked at non-government organizations (NGOs) or served as consultants and administrative staff catering to IDPs.

The researcher conducted FGDs to examine IDPs’ experiences of armed conflict and forced displacement and views on gender-based violence and security risks. The participants were identified through referrals of duty-bearers, particularly Peacekeepers and Job Order personnel in Zamboanga and NGO workers in Iligan. Separate FGDs were conducted with female and male adults and youth at traditional evacuation centers in Zamboanga and Iligan, transitory sites in Zamboanga, and a home-based evacuation community in Iligan. To ensure confidentiality, the participants were briefed about the need to refrain from disclosing the FGD proceedings; the non-disclosure clause was included in the consent form.

The researcher conducted 24 FGDs in Zamboanga; of these, eight FGDs were conducted at the Grandstand, and 16 FGDs at four transitory sites, namely: Buggoc, Masepla, Rio Hondo (see Table 2). Four FGDs were conducted with parents and four with youth at the Grandstand (for a total of 8). In each transitory site that the researcher visited, two FGDs were conducted with parents and two FGDs with youth (for a total of 16). The FGD participants in Zamboanga included 122 parents and 104 youth, who resided either at the Grandstand or transitory sites. The participants mainly identified with the Tausug and Sama-Badjao ethnic groups, which are affiliated with the Muslim community in the Philippines, and belonged to low-income and working-class communities. The FGDs typically had 6 to 10 participants. However, there were cases of low turnout. For instance, only four male youth participated at an FGD in the Grandstand. There were also instances wherein the turnout exceeded 10 to 12 participants; in one transitory site, 14 people participated at an FGD for male youth.

**Table 2:** Focus group discussions in Zamboanga City.

Location of focus group discussion (FGD)	Number of focus group discussions (FGDs) conducted	
Traditional evacuation center	FGDs with female participants	FGDs with male participants
JPE Grandstand	3 FGDs with mothers^1^ 2 FGDs with female youth	1 FGD with fathers 2 FGDs with male youth
Total number of FGDs at traditional evacuation center, disaggregated by sex	5 FGDs with female participants	3 FGDs with male participants
Total number of FGDs at traditional evacuation center	8 FGDs	
Government-funded transitory sites	FGDs with Female Participants	FGDs with Male Participants
Buggoc	1 FGD with mothers 1 FGD with female youth	1 FGD with fathers 1 FGD with male youth
Kasanyangan	1 FGD with mothers 1 FGD with female youth	1 FGD with fathers 1 FGD with male youth
Masepla (also called Mampang)	1 FGD with mothers 1 FGD with female youth	1 FGD with fathers 1 FGD with male youth
Rio Hondo	1 FGD with mothers 1 FGD with female youth	1 FGD with fathers 1 FGD with male youth
Total Number of FGDs at transitory sites, disaggregated by sex	8 FGDs with female participants	8 FGDs with male participants
Total number of FGDs at transitory sites	16 FGDs	
Total number of FGDs at traditional evacuation center and transitory sites, disaggregated by sex	13 FGDs with female participants	11 FGDs with male participants
Total number of FGDs at traditional evacuation center and transitory sites	24 FGDs	
Number of FGD participants across type of evacuation arrangement or temporary housing arrangement	122 parents 104 youth	

^1^The FGD with mothers had more than the expected number of participants, leading the researcher to conduct an additional FGD with this participant group. Meanwhile, in the first FGD that the researcher had intended to conduct with the fathers, no one showed up due to conflicts with their work schedule.

The researcher conducted eight FGDs in Iligan City, where IDPs from Marawi commonly evacuated; of these, four FGDs took place at Maria Cristina Evacuation Center, a traditional evacuation center, and four at a home-based evacuation community in Barangay Sta. Elena (see Table 3). Two FGDs were conducted with parents and two with youth at the traditional evacuation center. Two FGDs involved parents and another two included youth at the home-based evacuation community. The FGD participants in Iligan included 64 parents and 56 youth, who resided either in a traditional evacuation center or in a home-based evacuation community. The participants mainly identified with the Maranao tribe, an ethnic group in the Muslim community in the Philippines; others belonged to other ethnolinguistic groups originally from the southern Philippines, such as Visayan. While they were predominantly low-income and working class, a few were middle class, but vulnerable due to displacement. The FGDs typically had 10 to 15 participants. More parents participated in the FGDs at the home-based evacuation community, as compared to the traditional evacuation center. There was a high turnout for female youth participants at the FGDs at both the traditional evacuation center and the home-based evacuation community.

**Table 3:** Focus group discussions in Iligan City^1^.

Location of focus group discussion (FGD)	Number of focus group discussions (FGDs) conducted	
Traditional evacuation center	FGDs with female participants	FGDs with male participants
Maria Cristina evacuation center	1 FGD with mothers 1 FGD with female youth	1 FGD with fathers 1 FGDs with male youth
Total number of FGDs at traditional evacuation center, disaggregated by sex	2 FGDs with female participants	2 FGDs with male participants
Total number of FGDs at traditional evacuation center	4 FGDs	
Home-based evacuation community		
Barangay Sta. Elena^2^	1 FGD with mothers 1 FGD with female youth	1 FGD with fathers 1 FGD with male youth
Total number of FGDs at traditional evacuation center, disaggregated by sex	2 FGDs with female participants	2 FGDs with male participants
Total number of FGDs at traditional evacuation center	4 FGDs	
Total number of FGDs at traditional evacuation center and home-based evacuation community, disaggregated by sex	4 FGDs with female participants	4 FGDs with male participants
Total number of FGDs at traditional evacuation center and home-based evacuation community	8 FGDs	
Number of FGD participants across type of evacuation arrangement	64 parents 56 youth	

^1^This was a common evacuation destination for IDPs from Marawi City. ^2^*Barangay* is a Filipino word that means village. “Sta.” is the abbreviated version of the Filipino word “*Santa*,” which means saint.

Given the sensitivity of the issues discussed, resources were available in case the informants got triggered. In both locations, the interviews and FGDs were conducted within close proximity of duty-bearers who could provide psychosocial support, as needed. In Zamboanga, the interviews and FGDs were conducted at the Command Post, the WFS, or at a residence near duty-bearers, such as the Camp Managers^4^, Peacekeepers, and WFS Facilitator. In Iligan, the interviews and FGDs were conducted also within close proximity of duty-bearers, such as NGO staff, volunteers trained in stress debriefing, and military personnel.

The interviews and FGDs were transcribed; notes were also taken. The data were analyzed through manual and focused coding via Microsoft Word. To identify common themes in the informants’ narratives, the researcher utilized thematic analysis, an approach to qualitative data analysis that involves determining and assessing patterns in datasets (Braun and Clarke, 2012).

### Socio-demographic profile of survivors of gender-based violence

#### Informants from Zamboanga

The majority (34 out of 35) of the informants from Zamboanga are women. One informant is a man.

The majority (30 informants) are Muslims and/or identify as adherents of Islam. A Sama-Badjao informant in Zamboanga described her ethnic group as “Islam,” but not “Muslim”; this can be attributed to the differential meanings and stereotypes ascribed to ethnic groups associated with Muslim culture, and tensions between tribes. Other informants recognized the similar roots of the Sama-Badjao and the Sama-Bangingi communities, but maintained that they constituted different tribes. One informant reverted to Islam after marrying a Muslim of Tausug descent.^5^ Five informants were Christians, specifically Catholic.^6^

The informants come from diverse racial and ethnic minority groups in Philippine society (see Table 4). Their ethnic profile reflects their religious affiliation. The majority (21) of the informants belonged to Islamized ethnic groups^7^, such as Sama-Badjao (8 women), Sama-Bangingi (1 woman), and Tausug (12 women). Six informants identified with Christianized ethnolinguistic groups^8^, such as Visayan (4 women and 1 man) and Waray (1 woman). The remainder (8 women) were of mixed descent. One woman was of Chinese and Sama-Bangingi ancestry. Five identified with a combination of Islamized and Christianized ethnic/ethnolinguistic groups, such as: Sama-Badjao and Ilocano (1 woman), Tausug and Ilocano (1 woman), and Tausug and Visayan (3 women). One woman traced her ancestry to two Christianized ethnolinguistic groups, specifically Chavacano and Visayan. Another identified with two Islamized ethnic groups, namely Sama-Badjao and Tausug.

**Table 4:** Racial and ethnic background of interview participants from Zamboanga.

Racial and ethnic/ethnolinguistic group	Number
Islamized ethnic group	21
Sama-Badjao	8
Sama-Bangingi	1
Tausug	12
Christianized ethnolinguistic group	6
Visayan	5
Waray	1
Mixed descent (more than one racial or ethnic/ethnolinguistic group)	8
Chinese and Islamized ethnic group (Sama-Bangingi)	1
Islamized and Christianized ethnolinguistic groups	5
Sama-Badjao and Ilocano	(1)
Tausug and Ilocano	(1)
Tausug and Visayan	(3)
Two Christianized ethnolinguistic groups	1
Chavacano and Visayan	(1)
Two Islamized ethnic groups	1
Sama-Badjao and Tausug	(1)
Total	35

#### Informants from Marawi

The majority (21 out of 25) of the informants from Marawi are women. Four are men.

The majority (19 informants) are Muslims. The remainder (6 informants) are Christians, including Catholics and other Christian denominations.

The informants belong to minority racial, ethnic, and religious communities, including Islamized and Christianized ethnic/ethnolinguistic groups^9^ (see Table 5). The majority (15 informants) identified as Maranao, an Islamized ethnic group. A small number belonged to Christianized ethnolinguistic groups, such as Visayan (4 informants) and Waray (1 informant). The remainder (5 informants) were of mixed descent. Four identified with a combination of Islamized and Christianized ethnic/ethnolinguistic groups, such as: Maranao and Tagalog (1 informant), Maranao and Visayan (1 informant), and Maranao and Waray (2 informants). One informant identified with two Christianized ethnolinguistic groups, specifically Ilocano and Visayan.

**Table 5:** Racial and ethnic background of interview participants from Marawi.

Racial and ethnic/ethnolinguistic group	Number
Islamized ethnic group	15
Maranao	15
Christianized ethnolinguistic group	5
Visayan	4
Waray	1
Mixed descent (more than one racial or ethnic/ethnolinguistic group)	5
Islamized and Christianized ethnic/ethnolinguistic groups	4
Maranao and Tagalog	(1)
Maranao and Visayan	(1)
Maranao and Waray	(2)
Two Christianized ethnolinguistic groups	1
Ilocano and Visayan	(1)
Total	25

## Results and discussion

### Pathways to evacuation of internally displaced people

The researcher examined the pathways to evacuation of IDPs, as these shaped their specific experiences of and/or vulnerability to GBV in times of forced displacement due to armed conflict. The IDPs from Zamboanga and Marawi had two pathways to evacuation: traditional evacuation centers and home-based evacuation. The former involved inclusion in the Department of Social Welfare and Development’s list of IDPs in evacuation centers. The latter involved residing with family members, relatives, in-laws, and/or friends, or renting an apartment or a room. Cultural factors, based on ethnicity and religion, influenced their evacuation pathways. Both pathways were associated with particular vulnerabilities and risks for GBV in times of forced migration and displacement.

#### IDPs from Zamboanga

Residing in traditional evacuation centers was a more common pathway among IDPs from Zamboanga, who stayed at the Grandstand, Cawa-Cawa Boulevard, and a classroom at a nearby public school—implying the disruption of children’s education due to armed conflict. Some opted for home-based evacuation, such as renting a room or living with relatives, but it was not as common among the informants. Relocation to transitory sites or other temporary housing—or in limited cases, permanent housing—occurred after the clearing of the boulevard by the Zamboanga City Local Government Unit (LGU) and/or after the closure of Grandstand in 2015. Only one informant, whose home was partially damaged, was able to return to her permanent residence.

Cultural dynamics influenced residence patterns at the traditional evacuation center. The IDPs preferred to live alongside members of their tribe. Friction between the Tausug and Sama-Badjao people impacted their living situation. Others claimed the Sama-Badjao way of life—particularly their reliance on boats for their livelihood—accounted for their residence along the boulevard, overlooking the sea, as opposed to living in the Grandstand. People also lived alongside their ethnic group at the transitory sites. Some communities had predominantly Sama-Badjao residents, while others had predominantly Tausug residents.

#### IDPs from Marawi

None of the informants from Marawi resided in a traditional evacuation center during the research. They opted for home-based evacuation by residing with in-laws, relatives, and/or friends, or renting an apartment or a hotel room, albeit with many relatives.

The researcher interviewed a family who lived in a home-based evacuation arrangement near a traditional evacuation center in the municipality of Balo-i, Lanao del Sur. Their household included: an elderly couple, their two daughters, and the husband and four children of the younger of the two sisters. Their home, located in “Ground Zero” of the siege, was heavily damaged by the airstrikes and clearing operations of the military and the police. The older sister considered staying at a traditional evacuation center; having been released from prison in Manila less than 2 months before the conflict, she was indigent, although she occasionally received support from her partner, who remained incarcerated. She wanted to be with her family and worried about their health and safety if they would stay at the evacuation center. The younger sister, who was then 5 months pregnant, also avoided the evacuation center, fearing the rapid spread of diseases. Their entire family thus opted for home-based evacuation.

Some informants discussed the limitations of living with relatives or friends. They commonly asserted: “It’s different living in your own house.” Another informant shared that she and her children, including an infant, shared an apartment with at least 15 relatives. Because she worked as a guidance counsellor, while her relatives did not have steady employment, she shouldered many of their living expenses.

Some informants did not need housing, since they resided in other municipalities in Northern Mindanao or maintained a separate house, apartment, or room in Iligan, Cagayan de Oro, Balo-i, and other nearby cities or municipalities before the siege. Economic displacement was more applicable to them. They faced additional constraints and security risks due to working in Marawi during the conflict.

The informants’ accounts revealed the role of culture in determining IDPs’ residence in traditional evacuation centers or home-based evacuation arrangements. Most people displaced by the Marawi Siege identify as Maranao or have assimilated to Maranao culture. Emphasizing the cultural norm of *marhatabat* (honor or pride), some IDPs stated that it is shameful to let one’s relatives live in an evacuation center: “People look at you as having failed in your obligations as a relative.” According to some duty-bearers, IDPs in traditional evacuation centers had nowhere to go, lacked support systems, and/or were more indigent than home-based evacuees. In contrast, some parents who were home-based evacuees noted that they had limited provisions, as the government prioritized those in evacuation centers.

Prolonged displacement affected some informants. Some members of the family in Balo-i, including the parents and the older sister, relocated multiple times, be it to other parts of Mindanao or Metro Manila and provinces in Luzon, during and after the siege.

### Experiences of gender-based violence

The experiences of IDPs from Zamboanga and Marawi show that GBV exists on a continuum from personal violence to community-based and/or state-sponsored violence during war and conflict. While gendered violence—be in the private or public sphere—cuts across multiple social backgrounds, forced displacement due to armed conflict heightens people’s vulnerability. As the succeeding sections illustrate, IDPs from Zamboanga experienced numerous forms of interpersonal, community, and/or state-sponsored violence, during and after the siege. IDPs from Marawi also experienced various forms of community and state-sponsored violence during the siege. Low-income and working-class individuals, across different ethnic/ethnolinguistic groups, had limited safety nets and support systems. Inadequate resources and neglect by government authorities exacerbated people’s vulnerability.

The IDPs’ narratives of gender-based violence revealed common themes. Intersectionality was apparent in their experiences, in that their interconnected social positions, relating to gender, sexual orientation, ethnicity, religion, social class, age, and ability, made them vulnerable to specific forms of GBV and facilitated or constrained their access to safety nets and interventions.

### Gender-based violence in the family

There were nuances in the accounts of gendered violence in the private sphere by IDPs from Zamboanga vis-à-vis those from Marawi. The former were more open than the latter, who dealt with cultural norms constraining both the disclosure of domestic violence and the expression of emotion and vulnerability, particularly to networks outside of the family unit, which altogether informed the possibility of withholding information about personal violence in times of forced migration and displacement.

IDPs from Zamboanga revealed their experiences of interpersonal violence. Domestic violence was common. The majority (26 women) disclosed their experiences of abuse by their current and/or former husbands. Seven women experienced various forms of abuse by their current and former husbands, including physical abuse, marital rape, drug use, infidelity, and desertion. Their current or estranged spouses commonly became (more) violent after the siege.

Other IDPs endured other forms of interpersonal violence by other family members, relatives, and significant networks. One woman was physically and verbally abused by her drug-addicted brother. Another woman lost her husband to *rido* (clan feud or clan war among Muslims in the Philippines). One informant identified her aunt as the perpetrator.

Seven women experienced violence from multiple perpetrators, men and women alike. One informant was hit by her husband and her son, who were both addicted to drugs. For another informant, the perpetrators included her first husband and her sister, who had an affair, and her second husband’s family. One woman was abused by her mother and her husband, and another by her husband and his cousin. One woman reported abuse by her husband and her mother-in-law, and another by her mother-in-law and her sisters-in-law. One informant identified her mother, her husband, her uncle, and her cousins as the perpetrators.

Some informants’ accounts reflected the internalization of oppression and of cultural norms reinforcing gendered violence. For example, a woman of Sama-Badjao and Ilocano descent narrated how her husband raped her at the Grandstand: “He held a knife to my throat…I did not want my children to wake up, so I just let him have his way.” She contrasted the expected responses of “Christian” (Christianized ethnolinguistic groups) and “Badjao” (affiliated with Islam) women to marital rape: “For you Christians, you can say no. For us Badjao, we cannot.” Meanwhile, a woman of Visayan and Tausug descent downplayed the abuse of her second husband, who used drugs: “My husband has hit me many times. Even before the siege. I’d be lying if I said it was just once or twice. But it’s the kind of slapping that’s done out of love. Not too strong.”

Survivors’ accounts of personal violence reflect entrenched gender norms in Islamized ethnic groups, labeled as “Muslim culture,” extending to the practices of the Tausug, Sama-Badjao, and Sama-Bangingi communities, and Philippine society, that reinforce women’s submissiveness and men’s dominance, deference to parents and other authority figures, the status of being married, especially to prominent individuals or clans, and the stigma of a “broken family” (family with separated parents) or of rebelling against family members. These dynamics intensified people’s vulnerability to GBV.

In contrast, there were no accounts of personal violence among IDPs from Marawi. However, some duty-bearers mentioned reports about domestic violence in evacuation centers. During an FGD, some adolescent Maranao and Visayan girls acknowledged that couples f ought because of the lack of privacy for intimacy in the evacuation center. Multiple informants acknowledged the Maranao cultural norm of secrecy about domestic violence and the preference to settle this within the family, which could have precluded disclosures thereof. Another factor that amplified the non-disclosure of domestic violence was the Maranao cultural norm of withholding emotions and concealing vulnerability outside the family unit, even in times of conflict, as acknowledged by some informants.

### Gender-based violence in the community

The community is also a site for gendered violence, such as violent actions perpetrated in public and/or shared spaces and conflict zones (Aulette and Wittner, 2011; Giles and Hyndman, 2004; Johnson, 2009). Community-level GBV is unique in times of forced migration, distinct from other situations that are not characterized by armed conflict and displacement. The accounts of IDPs from Zamboanga and Marawi reflect this trend.

IDPs in Zamboanga shared multiple instances of community-based GBV. The informants confirmed the prevalence of child and adult prostitution and marital rape after the siege. Other forms of gendered violence persisted at the Grandstand. Women and girls experienced voyeurism, sexual harassment, and rape in public toilets, catcalling, physical abuse by other women IDPs while lining up for provisions such as water and food packs, and other daily forms of degradation, such as taking a bath in front of neighbors. These risks compelled mothers to accompany their daughters to public toilets. Those who were single were compelled to marry after acts of voyeurism, framed as a norm in Muslim culture “to restore dignity.”

Adolescent and young adult men were vulnerable to bullying, organ trafficking, and sexual harassment by gay men. Some expressed sentiments mirroring internalized oppression: “We’re men, anyway. We have nothing to lose.” Moreover, men were vulnerable to recruitment into extremist groups under false pretenses, such as being sent to the nearby province of Sulu, a hotspot for extremism, to claim relief goods supposedly sent from Brunei, but never returned.

Multiple marginalization made certain IDPs more vulnerable to gendered violence and impunity. Duty-bearers such as Peacekeepers, WFS Facilitators, and residents cited the gang rape of a Tausug speech-impaired gay man, who was in his early 20s, at a public toilet at the Grandstand. His positionality due to his gender, sexuality, ethnicity, religion, social class, and disability, amplified his vulnerability to gendered violence. Yet some adolescents took his victimization lightly. During an FGD, some teenage Tausug girls laughed while recounting the incident—particularly how he was sodomized with a bottle. During another FGD, some Tausug adolescent gay men, despite recognizing their marginalization based on their sexual orientation, were dismissive of his plight as a survivor of gang rape and joked about his inability to identify the perpetrator. A gay informant recalled: “He pointed to all the good-looking guys at the Grandstand.” Such responses reflect culturally-ingrained homophobia in the Tausug community and in Philippine society as a whole.

IDPs remained vulnerable to community-based gendered violence at transitory sites in Zamboanga. For women and girls, the risk of sexual harassment and rape persisted in these communities or at the outskirts. Teenage girls endured bullying in school and by outsiders after a controversial report surfaced about high school-age girls consenting to “*Tira bente* (sex for 20 pesos)” at a transitory site.

Some acts of violence were also racialized. Sama-Badjao youth of all genders reported victimization by rival ethnic groups. Young Sama-Badjao men, having been socialized to avoid confrontation, were vulnerable to *kursunada* (bullying or extortion), street fights, and muggings perpetrated by Tausug residents at the outskirts of transitory sites. Sama-Badjao students constantly reported being bullied by Tausug schoolmates, who challenged them to fight outside school despite—and perhaps because of—Sama-Badjao cultural values such as pacifism and non-violence. Some adolescent Sama-Badjao women informants disclosed their experiences of bullying by their Tausug counterparts in school—and internalized their oppression: “They say we are low-ranking because we are Badjao. It’s true anyway.”

Women’s capability for violence against other women and men was evident at the Grandstand and transitory sites in Zamboanga, which illustrates the extent to which women were not simply victims, but also perpetrators of certain forms of community-based GBV in times of conflict and post-conflict. This indicates that women perpetrators had internalized the societal acceptance of GBV that they would not hesitate to carry out against other women and men under conditions of forced migration and displacement. Some women informants experienced violence by other women, including the mistress of one woman’s husband, neighbors, and even strangers—in the case of those victimized by other women IDPs while lining up for water—at the Grandstand. In other cases, informants were both a survivor and a perpetrator of abuse. A woman, who was victimized by her sister and her in-laws, admitted to chasing after her common-law partner with a knife at a transitory site, claiming that his refusal to marry her worsened her in-laws’ disdain, given the taboo on cohabitation in the Muslim community.

In contrast, IDPs from Marawi did not disclose many instances of community-based GBV. Some duty-bearers received unconfirmed reports about domestic violence in evacuation centers. The youth acknowledged occasional fights between couples, related to financial matters and men’s frustrations about not being able to have sex with their wives due to the lack of privacy. Yet a code of secrecy about domestic violence pervades Maranao culture, which poses challenges in ascertaining whether it does occur at the community level as part of the fights between couples, as witnessed in evacuation centers.

There were no known cases of child or adult prostitution among IDPs from Marawi. However, one duty-bearer mentioned an anecdotal, unconfirmed report about a woman “offering herself” in exchange for money due to hardship.

Some informants expressed misgivings about the absence of gender segregation in traditional evacuation centers, leading young women to interact frequently with men or to be seen in their regular activities. They claimed that shared spaces of women and men in evacuation centers are a taboo in Maranao culture, which stipulates the protection of daughters, who could only be seen by their parents, other family members, and other significant networks.

The risk of voyeurism in restrooms in traditional evacuation centers also surfaced. Like what the Zamboanga-based informant disclosed about “Muslim cultural norms” regarding marriage to restore dignity after cases of voyeurism, an informant from Marawi mentioned that in Maranao culture, a single woman would be expected to marry under such circumstances because “Her body parts have already been seen,” implying marriage as a remedy. The same informant disclosed that the Maranao community traditionally punished rape through public corporal punishment, by tying the perpetrator to a horse and dragging the offender around, as a deterrent.

### Gendered state-sponsored violence

The state is culpable of reinforcing and condoning gendered violence within and outside its borders, during and after armed conflict (Enloe, 2000; Human Rights Watch, 2007; Lee-Koo, 2011). The experiences of IDPs in Zamboanga and Marawi reflect this, but with nuances in terms of the liability of state agents or extremists. For IDPs from Zamboanga, the perpetrators were extremists, while for IDPs from Marawi, the perpetrators included both extremists and state agents, such as military and police forces. The positionality of non-state agents, such as extremist groups, and state agents, such as the military and the police, also shows nuances in the types of GBV perpetrated against IDPs.

In Zamboanga, five informants and their children were held hostage by the MNLF during the siege. Two informants are sisters and disclosed that they and/or their children were shot. One woman lost her son. The other claimed that the bullet nicked her; her son was treated, but her daughter still has a bullet lodged in her body. None of them received government assistance, as they were home-based evacuees who rented a room instead of evacuating to the Grandstand; they subsequently moved to a faith-based temporary housing community.

During a follow-up visit, a leader at the said community, who worked as a WFS Facilitator at the Grandstand and as Job Order Staff at a transitory site, disclosed covert attempts by extremists to recruit residents of Zamboanga. Months before the conflict in Marawi, she noticed flyers advertising work for people who were computer-literate and willing to relocate to other parts of Mindanao. Her daughters attended an information session about the job. She advised them to leave when they were asked to relocate to northern and central Mindanao, which was linked to the activities of the Maute Group and ISIS.

Meanwhile, informants from Marawi related security and safety issues that reveal their vulnerability to state-sponsored GBV and militarism. Women and men IDPs from Marawi had untoward encounters with ISIS members and military personnel. For instance, ISIS fighters stopped one informant, who was 4 months pregnant, while she was evacuating with her young children, compelling her to recite Islamic prayers to avoid being shot. She and her children lay face-down on the ground, while a soldier was shot several feet from them. She also experienced harassment and verbal abuse at gunpoint when rescued by Islamophobic military personnel.

The women narrated the risk of gendered violence or secondary violence due to ties with family members or relatives, who were often men. Some ensured the safety of family members or relatives who were men, elderly, and at-risk, which illustrates the security risks confronting women due to their gendered relational roles. During an FGD, one woman shared that she outwitted ISIS members by offering them socks and other provisions, to ensure that her husband would not be taken and forced to fight with them. Another woman, who was a university administrator, witnessed ISIS fighters patrolling their street when she rescued her children and her elderly mother. She guarded her teenage sons because of the high risk of their abduction and forced membership in ISIS because of their gender and age. A young woman disclosed that she secured the safety of everyone in the Marawi City LGU, including then-Mayor Majul Gandamra, who was a highly-valued target, and her sister, who was 8 months pregnant.

Men informants from Marawi also experienced gender-based violence. An elderly Muslim-Maranao man was stopped by military personnel, who suspected that he was an ISIS supporter due to his clothing. During the FGD, a young Muslim-Maranao man disclosed that his colleague and best friend, a Christian, was shot dead by ISIS fighters for being unable to recite the Surah-Al-Fatihah. He recalled: “I told them to take me, too, but they refused. I wish I could forget about that experience.”

Some informants from Marawi disclosed that IDPs faced gender-specific risks for state-sponsored violence. The women acknowledged the threat of rape during the siege. This aligns with research about the weaponization of rape and other forms of sexual assault during armed conflict (Medica Mondiale, 2025; United Nations Office of the High Commissioner for Human Rights, 2025; Wali et al., 1999). Some criticized the rape jokes of President Duterte, who “gave” soldiers the right to rape women and assured them that he would back them up (Al-Jazeera, 2017; Selk, 2017); one woman, however, stated that the media took the joke out of context. Another significant issue was harassment by military personnel, who made Muslim women remove their hijab and other traditional covering at checkpoints and made eye contact with or heckled at them, in violation of Maranao cultural practices concerning the treatment of women. One informant claimed that her teenage niece and their family employee were harassed at a security checkpoint, which distressed their parents. Such narratives reveal the gender dimensions of violence and militarization in a conflict zone.

Men from Marawi faced the risk of forced membership in extremist groups, similar to their counterparts from Zamboanga. Abducting and/or forcing people to fight alongside extremists constitutes an act of GBV. The threat of retaliatory violence is an additional risk for those who resist and refuse to join extremist groups. The brother-in-law of one informant was taken by ISIS members; the mayor negotiated his release.

Other men, especially those wearing religious clothing, experienced harassment by the military and the police, be it due to the suspicion of their support for extremists or as a punishment for violating policies on curfew that were imposed as part of Martial Law in Mindanao. During the FGDs, at least five adolescent and young adult men shared that they were arrested for missing curfew, were humiliated and/or hit by the police, and rendered community service before their release.

Those working in Marawi during the siege dealt with multiple security risks. A woman faculty member of Mindanao State University asserted: “Anything can happen.” Two other women instructors disclosed that the issuance of a safe conduct pass was not a guarantee of their safety, given the threat of being hit by stray bullets and internal explosive devices (IEDs).

IDPs from Marawi voiced their misgivings about “not knowing who the real enemy is” and were divided in seeing ISIS as allies or enemies. During the FGDs with male youth, one participant mentioned that ISIS members intercepted the sack of rice he had brought when he and his family evacuated. Yet another participant noted that ISIS rebels let him ride on their motorcycle when he evacuated, also carrying a sack of rice. For the woman held at gunpoint by ISIS insurgents^10^ and military personnel, she acknowledged that ISIS fighters helped her husband’s cousin and other civilians by giving them white shirts and teaching them escape routes during airstrikes. Other IDPs and duty-bearers asserted that ISIS members took advantage of Marawi residents’ resentment toward the government for the destruction of their homes and the climate of Islamophobia to recruit members.

Numerous informants cited the threat of clan feud, which involves killings perpetrated against entire families and clans upon returning to Marawi. Some Muslim-Maranao IDPs had openly vowed to exact revenge on anyone behind the siege, implicating their family members and relatives. The informants were divided on whether the victims of clan feud were limited to men, or if women could also be targeted. In any case, the risk of clan feud, with its potential lethal consequences, could lead to a vicious cycle of violence and retaliation involving violence, with gender dimensions. Whether it is only the men or both men and women of entire families and/or clans who are targeted, the repercussions of clan feud are evident, in terms of the dangers of becoming an unwitting victim of violent acts due to conflicts involving immediate and/or extended family networks. Clan feud, which often leads to unresolved killings, is a culturally-ingrained form of GBV, in that it predominantly victimizes men belonging to the family or clan targeted by the perpetrator. Outsiders and even law enforcement often hesitate to intervene due to the belief that it should be settled by the concerned families and clans, and the fear of being implicated in the violence. This trend is likewise evident in the narratives of some survivors of the Marawi Siege, who initially assumed that the gunfire they heard was simply connected to clan feud, only to find out that armed conflict was underway.

### Intersectionality in gender-based violence

The IDPs’ narratives show multiple, overlapping inequalities relating to race, ethnicity, religion, nationality, social class, gender, sexuality, and ability, which shape their vulnerability to violence. Intersectionality informed their experiences of GBV and other security risks. The impact of poverty and material hardship, compounded by displacement, on gender-based violence was evident in the accounts of IDPs from racial, ethnic, and religious minorities. Poverty was a cause of fights, leading to domestic violence, which intensified after armed conflict. For IDPs from Zamboanga, poverty was a barrier to leaving abusive marriages. For IDPs from Marawi, poverty and indigency informed people’s prolonged stays in evacuation centers. In both locations, forced displacement compounded people’s vulnerability and reinforced their limited resources and support systems in the face of gender-based violence. Middle-class individuals had more safety nets, as compared to their low-income and working-class counterparts.

Racial and ethnic beliefs and religious norms intersected with gender ideologies, influencing people’s responses or passivity to gendered violence. IDPs from Zamboanga, who belonged to or married into Islamized ethnic groups, faced cultural pressures for women to endure spousal abuse and avoid a “broken” family. In the case of the speech-impaired young adult Tausug gay man, who survived gang rape, entrenched religious and cultural homophobia and disability-related impediments, enabled the impunity of the perpetrators and the indifference of some people. For IDPs from Marawi, race and ethnicity, religion, and gender, influenced the code of silence about domestic violence because of the Maranao cultural belief that this should be handled within the family.

A form of collective violence against IDPs lies in their dismal living conditions many years after their displacement due to armed conflict. This reinforces their vulnerability to gendered violence, their experience of neglect due to limited interventions by government authorities in providing decent housing, livelihood, health care, and other basic needs, and their struggles for dignity and subsistence (Cepeda, 2017; InterAksyon.com, 2016).

Discussion: Implications of Findings.

The experiences of IDPs from Zamboanga and Marawi resonate with research on the gendered continuum of violence, ranging from personal violence between intimate partners, acquaintances, and strangers, to community-based and/or state-sponsored violence during war and conflict (Aulette and Wittner, 2011; Kelly 1988, cited in Radford and Stanko, 1996; Lorber, 2005; United Nations Development Fund for Women, 2003). The accounts of women IDPs from Zamboanga who had survived spousal, family, and/or other forms of personal violence, and even the cultural norm of secrecy regarding domestic violence among IDPs from Marawi, reveal intersecting social positions that influence GBV in the private sphere. This aligns with research findings about how family structures reflect gender expectations in specific cultures, which are shaped by social forces, such as sexism, racism, poverty, and homophobia (Disch, 2009; Radford and Stanko, 1996). Violence is thus a means to maintain power and oppression. In times of armed conflict and forced migration, gendered violence is a way of exerting control and dominance over IDPs who are already constrained by multiple vulnerabilities and disadvantages.

The narratives of IDPs from Zamboanga and Marawi illustrate that private and public acts of violence are interconnected; the violence confronting women and men in conflict zones is an extension of the culture of violence in their daily lives. This aligns with research about the links between violence in the private and public spheres (Caiazza, 2004; Marshall, 2006; United Nations Development Fund for Women, 2003; Wali et al., 1999). The experiences of IDPs also illuminate another dimension of the continuum of GBV—that is, the interrelationship between structural and interpersonal violence in times of displacement. This aligns with research confirming the continuum of structural and interpersonal gendered violence during forced migration (Hourani et al., 2021; Phillimore et al., 2025; Sisic et al., 2025).

The accounts of IDPs from both locations confirm that gender-based violence is intertwined with the culture of violence used to maintain systems of oppression in different communities (Disch, 2009; Kelly, 1996; Steinem, 2004). Gender privilege is ingrained in the operations of society, and the organization of social structures and institutions reflects and perpetuates “power over” another (Johnson 2009). Sexual violence enables men’s dominance and women’s subordination (Radford and Stanko, 1996; Kelly, 1996; Sheffield, 2004; United Nations Development Fund for Women, 2003; World Health Organization, 2006).

The narratives of IDPs from Zamboanga and Marawi align with research on how gender intersects with other statuses, such as race and ethnicity, religion, nationality, social class, sexuality, age, and (dis)ability, in impacting people’s vulnerability to gender-based violence (Davis, 1983; Davis, 1984; Hooks, 2000; Lerner, 1986; Radford and Stanko, 1996). At the same time, their stories present unique findings in terms of the lived experiences and particularities of IDPs during both conflicts. Their stories illustrate varying experiences of gender-based violence according to ethnic and religious groups in the Philippine context.

People’s position in the matrix of domination and privilege affects their experiences of victimization and the responses of the state, law enforcement, professionals, and volunteers (Barbee and Little, 1993; Disch, 2009; Green, 1999; Hester et al., 1996; Hill Collins, 2000; United Nations Development Fund for Women, 2003). Gender, sexual orientation, social class, race, ethnicity, religion, shape specific acts of violence against IDPs (Veloso, 2022a; Veloso, 2022b). Cultural norms, including secrecy, passivity, and fatalism in times of crisis shape the responses of IDPs and communities to gender-based violence. It is crucial to address internalized oppression and the normalization of gender and racial/ethnic inequality,

## Conclusion

The IDPs from Zamboanga and Marawi experienced forced displacement due to armed conflict. This research found two main pathways to evacuation of IDPs from Zamboanga and Marawi—residing in traditional evacuation centers and home-based evacuation communities. Intersecting social divisions influenced the pathways to evacuation of IDPs from both cities. Cultural factors, such as ethnic and religious background and social class, influenced residence in traditional evacuation centers or home-based evacuation communities. Both pathways were fraught with specific vulnerabilities and risks of gendered violence, in the particular context of forced migration and displacement.

Forced displacement, as a form of involuntary migration, frequently involves the risk of gender-based violence, which spans a continuum from personal to community-based and state-sponsored violence. The particular form in which forced displacement took place (“evacuation pathway”), shaped to a large extent by the social positionality of the survivors in each case, led to distinct risks and vulnerabilities depending on the pathway. IDPs from Zamboanga, who largely resided in traditional evacuation centers, experienced multiple forms of interpersonal, community, and state-sponsored violence during and post-conflict, while IDPs from Marawi, who commonly opted for home-based evacuation, experienced different forms of community and state-sponsored violence during the conflict. Their experiences show how the interconnections between gender, sexuality, race and ethnicity, religion, nationality, social class, age, and (dis)ability, among other social positions, shaped their experiences of displacement and gendered violence during armed conflict and/or its aftermath, and their access to safety nets and support systems or lack thereof in a postcolonial, low-income nation such as the Philippines.

IDPs endured numerous intertwined forms of private and public violence and of interpersonal and systemic or structural violence. The different forms of interpersonal, community-based, and state-sponsored gendered violence illustrate that violence is not only an individual strategy for maintaining control in relationships, but also a collective strategy used to reinforce hierarchies based on difference. Cultural and social norms influence how they frame their experiences of and responses to gender-based violence in the context of forced migration and displacement.

## Data Availability

The raw data supporting the conclusions of this article will be made available by the authors, without undue reservation.

## Footnotes

1 This region is now known as the Bangsamoro Autonomous Region of Muslim Mindanao.

2 For the informants in Zamboanga, the researcher obtained permission from the head of the Municipal Social Welfare and Development Office (MSWDO) before conducting key informant interviews (KIIs) and focus group discussions (FGDs) at the Grandstand. She also coordinated with the Women-Friendly Space (WFS) facilitator and the Camp Manager, Peacekeepers, and Job Order Staff at the Grandstand. The said duty-bearers referred her to all the interview and FGD participants. After the Grandstand closed, the researcher obtained permission from the head of the MSWDO before conducting KIIs and FGDs at the transitory sites. The researcher then coordinated with the Camp Manager and Job Order Staff at the transitory sites. The said duty-bearers also referred her to KII and FGD participants. The researcher also coordinated with the former WFS Facilitator, who had relocated to a faith-based temporary housing community but worked at a government-run transitory site, and referred her to other interviewees at both locations. Moreover, one of the residents at the faith-based housing community knew the Job Order Staff at some transitory sites and offered to participate in an interview.

3 For the informants from Marawi, the researcher first contacted people in her network who had evacuated from Marawi and/or worked in Iligan and served IDPs. Three informants referred her and helped connect her to other interviewees, including the director of a non-government organization (NGO). The said NGO then assisted her and accompanied her in coordinating with traditional and home-based evacuation communities in order to conduct FGDs. For the family in Balo-i, Lanao del Norte, the researcher met one of the daughters through prior volunteer work with persons deprived of liberty (PDL) in Manila and contacted her and requested to interview the family.

4 The designation of “Camp Manager” is the term was used to refer to the head of the Grandstand, a traditional evacuation center, and the transitory sites for IDPs in Zamboanga City. The management of both types of evacuation sites was overseen by a Camp Manager.

5 In Islam, some people prefer the term “revert” instead of “convert” because of the belief that humans are born with *fitra*, an innate knowledge of or natural disposition toward God; thus, a person who accepts Islam is “reverting” to this original state (El Alam, 2022).

6 In the Philippines, people are distinguished according to race, ethnicity, and religion. Race and ethnicity tend to overlap with religion because the Spanish and American colonization historically led to the institutionalization of racial categories, namely: Christian, Muslim, and indigenous peoples (IPs), depending on whether people’s ancestors had converted to Christianity and assimilated to colonialism, or embraced Islam or maintained their indigenous religion and thus resisted colonialism (Asian Development Bank, 2002; Ty, 2010). Even people’s ethnic heritage is associated with ethnic groups that had historically converted to Christianity or Islam or retained their ancestral religion (Berkley Center for Religion, Peace & World Affairs, 2013). The stereotypes that are associated with being a Christian and a Muslim, as well as an indigenous person, tend to overlap with racial and ethnic stereotypes.

7 To reflect the informants’ views accurately, the researcher has opted to use the term “Islamized tribes” or “Islamized ethnic groups” referring to their history of embracing Islam prior to the Spanish colonial era. In addition, the researcher uses “ethnic groups” and “ethnolinguistic groups” interchangeably.

8 To reflect the informants’ views accurately, the researcher has opted to use the term “Christianized ethnolinguistic groups,” referring to their history of converting to Christianity during the Spanish colonial era.

9 Please refer to the previous footnote about the overlap between race, ethnicity, and religion in the Philippines due to the country’s history of colonialism by Spain and the United States (Berkley Center for Religion, Peace & World Affairs, 2013; Ty, 2010).

10 The author used the terms “insurgents” and “fighters” interchangeably to refer to ISIS members.

## Author disclaimer

This article is culled from three descriptive, qualitative research projects about forced displacement in two conflict zones in the southern Philippines. Two studies focused on IDPs who were residing mainly in traditional evacuation centers and government-funded and faith-based transitory sites, as well as one who had relocated to permanent housing, in Zamboanga City. Another study focused on IDPs at a traditional evacuation center and home-based evacuation communities in both Iligan City and the municipality of Baloi, Lanao del Sur, as well as IDPs who had resumed work in Marawi City at the height of the conflict. This article brings together the material of the two aforementioned studies and is thus a progression from the author’s previous publications on gender-based violence during and after the Zamboanga Siege (Veloso, 2022a) and during the Marawi Siege (Veloso, 2022b). This paper includes the same research population of IDPs from Marawi, as discussed in the previous article (Veloso, 2022b). The current article includes an additional research population consisting of IDPs from Zamboanga City, who are from a different conflict zone, where the author also conducted research (Veloso, 2022a). This article compares and contrasts the pathways to evacuation of IDPs from Zamboanga and Marawi and how these shape the risks of GBV among survivors, and the trends in and risks for gender-based violence impacting these population groups, so it is different from the author’s previous publications, which examined the experiences of IDPs from the aforementioned conflict zones separately (Veloso, 2022a; Veloso, 2022b).
